# Supplementary material for: Effects of nutrient supply on leaf stoichiometry and relative growth rate of three stoloniferous alien plants
Source: PLoS One. 2022 Dec 2;17(12):e0278656. doi: 10.1371/journal.pone.0278656 (PMC9718409; doi:10.1371/journal.pone.0278656)
Supplement: Attachment — Submitted filename: comments.docx [file pone.0278656.s002.docx]

In the paper, the authors addressed the effects of nutrient supply on their leaf stoichiometry and relative growth rate. As I know, the researches on the correlationship bewteen leaf stoichiometry of alien clonal plants are scarce. In this sense, this manuscript is a very good paper. Additionally, the experiment is also well designed and results are also very interesting. However, there are a couple of points that will need to be added or improved.

Line 2, “change” should be “changes”.

Line 7-8， “leaf” should be “leaves”

Line 10，“level” should be “levels”。

Line 10 and Line 12，“correlationship” should be “correlation”。

Line 14， “may be play an important role….” should be “may play an important role….”or “maybe play an important role….”。

Line 20， “plant” should be “plants”。

Line 29， “similar pattern” should be “the similar pattern”.

Line 37， “such as microbe” should be “such as microbes”。

Line 49， “bewteen” should be “between”。

Line 81， “was applied in the experiment” should be “were applied in the experiment”。

Line 84， “pH of nutrient” should be “The pH of nutrient”。

Line 104-106， “The classification of H values is” should be “classifications of H values are”

Line 106-107, “it often is” should be “it is often”.

Line 106-107, “fit” should be “fitting”.

Line 146， Fig 1 “supply ” should be “supplies”。

Line 207-209，“decreased in plant subjected high” should be “decreased in plants subjected to high”.

Line 212, “Comparing with” should be “Compared with”.

Line 214, “N:P ratio in leaf of was” should be “N:P ratio in leaf was”.

Line 215-216, “more studies are need” should be “more studies are needed”.

Line 215-216, “between N:P ration” should be “between N:P ratio”.

Line 220-221, “presented positive influence” should be “presented a positive influence”.

Line 224, “growth rate of plant and its leaf N:P ratio” should be “growth rate of plants and the N:P ratio”.

Line 230, “nutrientsupply” should be “nutrients supply”.

Line 234, “Jinagxi Province” should be “Jiangxi”.

Line 240， “ may be play” should be “ may play” or “maybe play”.
